# Treatment of Pneumonia

**Published:** 1889-02

**Authors:** T. J. Bennett

**Affiliations:** Austin, Texas


### TREATMENT OF PNEUMONIA.

By T. J. Bennett, M. D., Austin, Texas.

[Read before the Travis County Medical Society.]

AT THE last meeting of this Society, Dr. B. F. Church read a paper entitled, "Is pneumonia an essential fever?" He discussed the history, pathology and etiology of the disease, particularly the latter, as based upon the germ theory, and the dry air and chloride of sodium theory recently advanced by Dr. Baker, of Michigan.

The paper was interesting and instructive, and with the discussion which followed the subject was brought well up to treatment, which latter was assigned to me for presentation this evening.

As other scientific therapeusis is based upon our knowledge of pathology, you will pardon me for referring briefly to this condition, as well as to the types of the disease under consideration.

Pneumonia is simply an inflammation of the lungs—that is, of the air cells and the alveoli. The types are the *catarrhal, croupous, and interstitial.*

Dr. Ferrand defines the word "catarrh" to mean "an elementary lesion of a secreting surface—a hyperæmia." The catarrhal form of pneumonia is characterized by an inflammation which invades first the bronchi, and then extends to the air cells. The exudation consists of epithelial cells and serum. It is not generally agreed that the inflammation in this form of pneumonia ever extends to the alveoli, nor that the exudation ever contains fibrin.

The inflammation in lobar, or croupous pneumonia, originates in the alveoli and air cells. The exudation is composed of epithelial cells, serum and fibrin, the latter being claimed as the chief differential feature in this form of pneumonia. The bronchi are effected only secondarily.

Blood corpuscles are present in the exudation of both forms of the disease.

With reference to the inflammation not involving the alveoli in one form of pneumonia and doing so in others, it seems improbable, since the air cells and alveoli are so closely related anatomically. It also seems improbable that an inflammation of the same degree in the same structure should produce an exudation of fibrin in one case and not in another.

The consolidation which is observed in both forms of the disease is due to the same cause, viz: to the exclusion of air which is displaced by the infiltration with newly formed cellular elements, the exudation.

The interstitial form of pneumonia consists in a hyperplasia of connective tissue, and may follow either the catarrhal or croupous form, or may develope gradually in company with tuberculosis.

The causes of pneumonia are not positively known. Sudden changes in the temperature of the air, exposure to inclement weather and over work constitute the chief causes as ordinarily observed.

I need not further notice this part of the subject, nor the question as to whether any form of pneumonia is an essential fever. Dr. Church's paper fully brought out the arguments on these points. I will say, however, that I believe pneumonia, under all circumstances, to be a local inflammation, a symptomatic disease, and capable of being aborted in the first stage, or stage of engorgement. This is accomplished by the judicious use of quinine and Dovers powders, or morphine and veratrum viride hypodermically, assisted by a hot foot bath and a sinapism over the seat of the pain. Abortive measures, however, must be brought to bear upon the case before the ephithelium of the air cells has been destroyed by the processes of inflammation, otherwise the effort at disengorgement would fail, since the damage has already been done. Once the denudation of epithelium and other destructive changes of inflammation completed, the disease assumes the course apparently of a specific ailment, and the treatment must necessarily be symptomatic.

The disease abates only when there has been time for repairs through the agency of nature and the art of man.

Then in the early stage of pneumonia, before destructive changes have taken place, I am an advocate of active medication, and I feel certain that I have been rewarded with splendid results. For instance, being called to a case which is merging from a chill (for a chill is common in the beginning), with high fever, pain in side, rapid breathing, lips cyanotic, pulse full and quick, and the other physical signs of acute pneumonia, I would at once direct ten or fifteen grains of Dovers powders, or give a hypodermic of morphine, one-fourth grain, and ten grains of quinine. Better than the quinine, I think, for controlling the circulation, is fluid extract of veratrum viride in eight or ten drop doses, repeated every three-quarters of an hour or hour until the pulse is softened and made to number about seventy to the minute. In addition to this, as I have already stated, I would direct a hot foot bath, and a mustard to be placed over the seat of pain. In such a case, the whole tissue of the lung is increased in volume, and is infiltrated with a serous fluid, while there is more or less transudation of blood through the distended vessels, as is evidenced by the brick-dust sputa.

There are good reasons for giving veratrum in acute pneumonia as also there is good authority for so doing.

Dr. Strand, during last year, has had occasion to praise this drug in the treatment of the early stage of pneumonia. He concludes from abundant observations at the bedside that holding the pulse between 65 and 70 veratrum not only saves a further invasion of pulmonary area, but it cuts short the engorgement and inflammatory process. He claims that it prevents a failure of the heart since 70 pulsations to the minute will not exhaust its power. This effect of the drug maintained hinders purulent inflammation, since this is the result of over action of the heart pumping more blood to the lung than the veins can return.

I am satisfied the chief value of veratrum "is confined to the influence it has in diminishing the blood-supply to the congested organ." After fibrinous exudation and hepatization have taken place, which is always after the destruction of epithelium, veratrum is of little or no service; in fact it may do great harm by depressing the patient.

Upon this same principle Petresco, an eminent French authority, in a late medical journal, reports wonderful success in the treatment of acute pneumonia with the infusion of digitalis. The infusion is prepared with one drachm of digitalis leaves to 50 drachms of water and ten drachms, of simple syrup. He gives one drachm every half hour, and says the disease is generally checked in three days. The fever and all the physical phenomena, local as well as general, disappear rapidly. In spite of the large dose Petresco has not seen poisonous effects, tolerance having been incontestably proved by 577 observations. By this treatment it is claimed the mortality of pneumonia has been reduced to 1.22 per cent.

A number of journal articles have recently appeared advocating and attempting to revive venesection in the treatment of pneumonia. I have had no experience with this method. I have always thought the patient needed all the blood he had to enable him to withstand this formidable disease, and that it would be better practice to control the circulating fluid rather than withdraw it.

It must be borne in mind always that the tendency to death is by heart failure.

The treatment of pneumonia, after it has passed beyond the stage of hopeful abortion, is largely symptomatic.

There are no specifics. Each case must suggest the remedies best suited to it. Supportive agents, not depressants, should be held uppermost throughout the management of all cases. If the temperature should rise above 103.°5 a dose or two of antipyrin should be administered and the fever reduced.

Dovers' powders or morphine should be given to control pain, and chloral sulfonal to produce sleep. A light flax poultice on the chest effects favorably the temperature, and is soothing to the patient.

Carbonate of ammonia is a remedy that is indicated in nearly all cases. It is a diffusible stimulant and most all authorities claim for it the property of hindering the fibrination of the blood which is the great tendency in pneumonia. It liquifies the exudation and promotes expectoration. The disagreeable taste is admirably covered up by the elixir of licorice.

Whisky or brandy is indicated when a stimulant is needed. Nothing will take its place when the vital powers are flagging. One tablespoonful of whisky every two or three hours for an adult will usually keep up a wholesome stimulation. I prefer small doses frequently repeated to larger ones given at longer intervals, because the effect of the latter is depressing rather than stimulating.

Equal parts of camphor water and liquor ammoniæ acetatis in tablespoonful doses often answers a good purpose as respiratory stimulant. Many other valuable preparation could be mentioned such as the iodide of ammonia and tartar emetic in the early stage.

The iodide of ammonia combined with terpina hydrate is an excellent prescription where resolution is slow, or in the interstitial form it is one of the best with which I am acquainted.

Many cases of pneumonia pass through the whole course of the attack with but little demand for any kind of medication. Conservatism is wise in such cases.

The complications, which arise during an attack of course, are to be met symptomatically.

Good nournishment is an important part of the treatment. The digestion should be protected by not over feeding. The bowels should be kept open. In short the patient should be treated as well as the disease.

DISCUSSION.

Dr. T. J. Turner, in referring to the pathology of pneumouia, said he did not believe it to be a specific infectious disease, but purely a local affection, and should be treated as such. Thought many cases could be aborted in the early stages by antiphlogistic measures, and, that the same indications for the use of opium prevailed in this disease as in peritonitis. That the extra work placed upon the heart to overcome the resistance in the pulmonic circulation during hepatization of a part of the lung area, was a cause of cardiac paralysis.

Dr. Q. C. Smith said no set rules could be laid down for the treatment of pneumonia. In the croupous form of the disease in a sthenic adult, he would not hesitate to bleed. He related the history of several cases that occurred in a malarious district which he aborted by first giving an emetic, and then aconite in small doses, frequently repeated, till the temperature was reduced, which was generally accompanied by diaphoresis. This was followed by quinine in moderate doses for several days. He reccommends and uses aconite for the conditions in pneumonia in which opium is generally considered to be indicated, and frequently derives all the benefit from the latter, minus its objectionable effects.

Dr. J. W. McLaughlin maintains the belief that croupous pneumonia is an essential fever, but does not hold this view of the lobular or catarrhal form. In mild cases of croupous pneumonia he thinks little treatment is required. The main conditions for treatment in any case is to control pain, and maintain the powers of the patient. Stimulate freely; not in favor of veratrum viride as he thinks it favors heart failure; prefers digitalis. The specific cause of the disease is the controlling factor in the causation of cardic paralysis. Would bleed in favorable cases. B. F. C.

